# Supplementary material for: A Comparison of the Genotoxic Effects of Gold Nanoparticles Functionalized with Seven Different Ligands in Cultured Human Hepatocellular Carcinoma Cells
Source: Nanomaterials (Basel). 2022 Mar 29;12(7):1126. doi: 10.3390/nano12071126 (PMC9000686; doi:10.3390/nano12071126)
Supplement: Supplementary file 1 [file nanomaterials-12-01126-s001.zip › nanomaterials-1580027-supplementary.pdf]

nanomaterials

MDPI

Article

A Comparison of the Genotoxic Effects of Gold Nanoparticles Functionalized with Seven Different Ligands in Cultured Human Hepatocellular Carcinoma Cells

Danielle Mulder *, Cornelius Johannes Francois Taute, Mari van Wyk and Pieter J. Pretorius

Human Metabolomics, Potchefstroom Campus, North-West University, Potchefstroom 2351, South Africa; ftaute2015@gmail.com (C.J.F.T.); 12791733@nwu.ac.za (M.v.W.); pietp484@gmail.com (P.J.P.)

* Correspondence: 26718944@g.nwu.ac.za, Tel.: + 27–018–299–2307

Table S1. Properties of capping agents used to functionalize the gold nanoparticles.

Ligand	Structure	Net charge at physiological pH	pKa	GNP pH stability	Application	Ref*
**BSA**	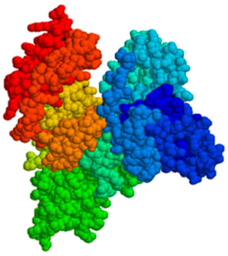	Negative (Bueno and Petri, 2014)	5.4	pH 7	Transport vehicles for proteins, hormones and diagnostic agents in biomedical field	Sigma (Nadi et al., 2014)
**Citrate**	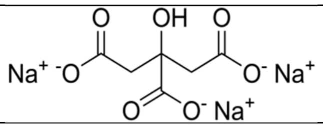	negative	3.138<br>4.76<br>6.40	-	Anti-coagulation	(Tolwani et al., 2001)
**GSH**	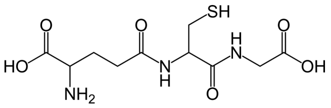	Negative (Okumura et al., 2012)	2.12<br>3.53<br>8.66<br>9.12	pH 8	Antioxidant	(Townsend et al., 2003)
**MUA**	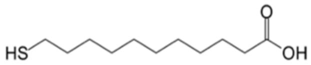	Negative (Simonian et al., 2002)	6.5	pH 6	Drug delivery	(National et al., 2015)
**PEG**	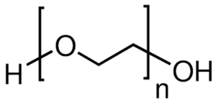	Neutral	16	pH 7	Tablet coating	Sigma (Gans and Chavkin, 1954)
**PSSNA**	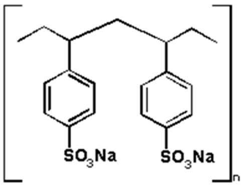	Negative<br><br>(Shovsky et al., 2012)	1-2	pH 6	Drug delivery	(Venkatesan et al., 2013)
**PVP**	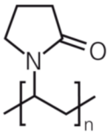	Negative	5	pH 5	Cosmetics, food and drug delivery	(Nair, 1998)

Table S2. Final concentration of ligand added to the gold nanoparticles for functionalization.

	pH of gold for ligand exchange	Final concentration
**BSA**	7	0.048mg/ml
**PEG**	7	0.33%
**PVP**	5	0.22mM
**PSSNA**	6	0.048%
**MUA**	6	0.057mM
**GSH**	8	1.19mM

Table S3. Final dosage concentrations (IC30) for Au-Ligands.

	WST-1 Dosage	Concentration (nm)	Concentration (µg/mL)	Molar mass (g/mol)
**BSA**	3 (430pM)	0.43	0.028	66 463 Da
**Citrate**	4 (860pm)	0.86	2.22×10^{-4}	258.07
**GSH**	2 (220pM)	0.22	6.27×10^{-5}	307.32
**MUA**	2 (220pM)	0.22	4.8×10^{-5}	218.36
**PEG**	3 (430pM)	0.43	2.15×10^{-3}	5000
**PSSNA**	3 (430pM)	0.43	0.086	200 000
**PVP**	3 (430pM)	0.43	0.0172	40 000

Calculation used for converting nm to µg/mL (Abcam, 2021)

nm concentration x Mw

1×10^6

Gel electrophoresis

The charge of the functional group was determined by performing an agarose gel electrophoresis assay. The positively charged particles will migrate to the anode of the gel and the negatively charged particles will migrate to the cathode. The samples which aggregated in the TBE buffer were then re-run in a TAE buffer.

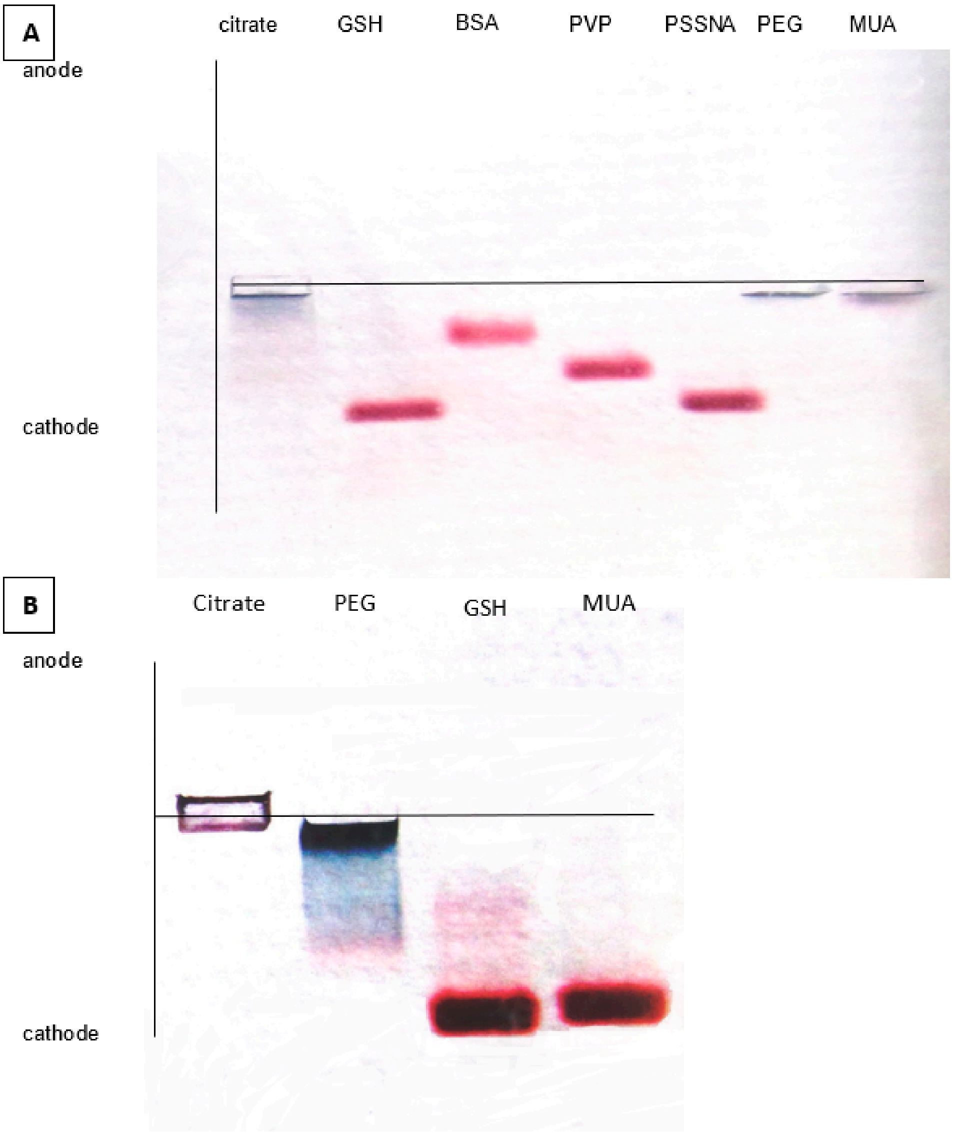

Figure S1. (**A**) Gel electrophoresis done in TBE pH8. (**B**) Gel electrophoresis done in TAE pH8.

Table S4. Stability of the particles in environments similar to those which were carried out during the cell biology section of the experiment.

**Compound type**	**Compound**	**Ligand**						
		**BSA**	**Citrate**	**GSH**	**MUA**	**PEG**	**PSSNA**	**PVP**
**Salt**	**1mM NaCl**	Stable	Stable	Stable	Stable	Stable	Stable	Stable
**Thiols**	**β-Mercapto-ethanol**	Unstable	Unstable	Unstable	Unstable	Unstable	Unstable	Stable
**Medium**	**Supplemented**	Stable	Stable	Stable	Stable	Stable	Stable	Stable
**Buffers**	**10mM Mops**	Stable	Stable	Stable	Stable	Stable	Stable	Stable
	**100mM Hepes**	Stable	Stable	Stable	Stable	Stable	Stable	Stable
	**0.01x EDTA**	Stable	Stable	Stable	Stable	Stable	Stable	Stable
	**1x PBS**	Stable	Unstable	Unstable	Unstable	Unstable	Unstable	Unstable
**pH**	**6-7**	Stable	Stable	Stable	Stable	Stable	Stable	Stable
**Organic molecules**	**20mM Glycine**	Stable	Stable	Stable	Stable	Stable	Stable	Stable
	**5.04mM citrate**	Stable	Stable	Stable	Stable	Stable	Stable	Stable

The salt environment and buffers such as Mops, Hepes, and EDTA and organic environments such as glycine and citrate were unproblematic. GNP-GSH was unstable in the supplemented medium. Most of the GNP-ligands were unstable in PBS buffer and β-mercaptoethanol. PBS was, therefore, not a good carrier to resuspend the GNP-ligands in once the samples had undergone ligand exchange and clean-up in preparation for cell culture. The β-mercaptoethanol was an indication that once the GNP-ligands were absorbed in the cell, possible competing thiolated molecules could displace the functionalized ligands.

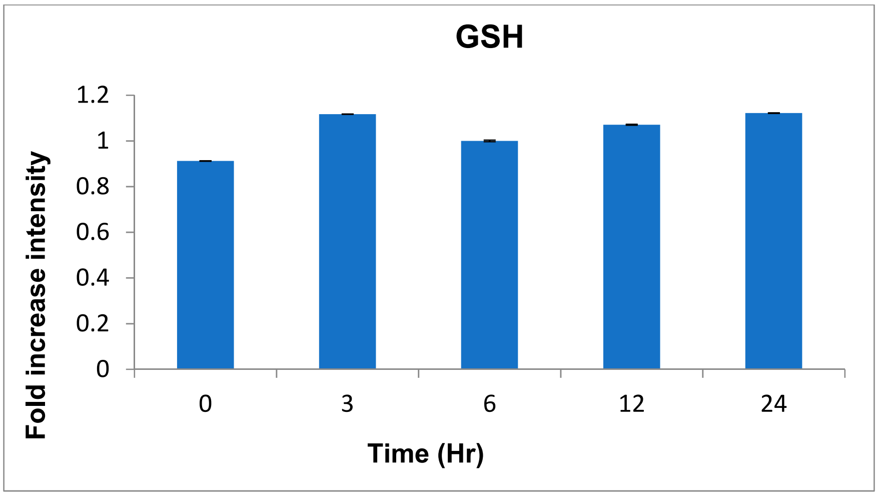
GSH
Fold increase intensity
1.2
1
0.8
0.6
0.4
0.2
0
0
3
6
12
24
Time (Hr)

(a)

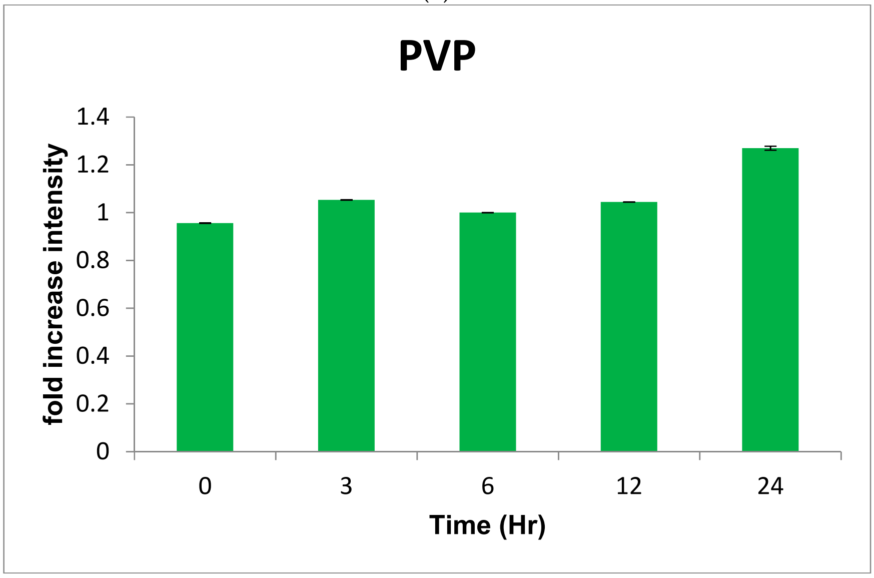
PVP
fold increase intensity
1.4
1.2
1
0.8
0.6
0.4
0.2
0
0
3
6
12
24
Time (Hr)

(b)

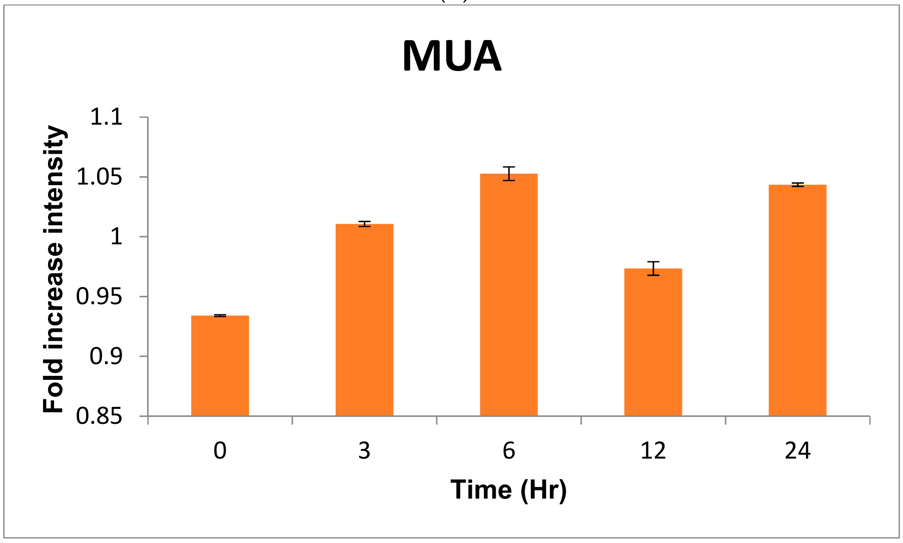
MUA
Fold increase intensity
1.1
1.05
1
0.95
0.9
0.85
0
3
6
12
24
Time (Hr)

(c)

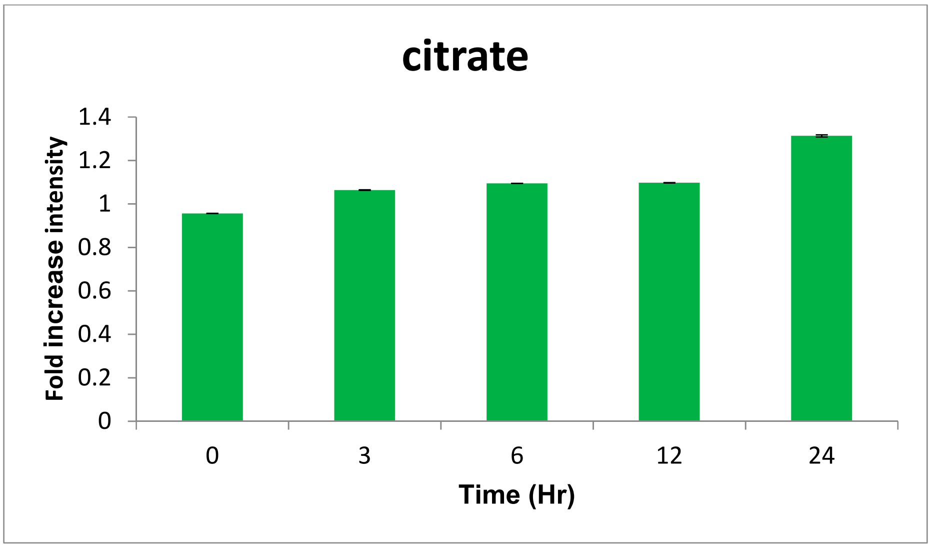
citrate
Fold increase intensity
1.4
1.2
1
0.8
0.6
0.4
0.2
0
0
3
6
12
24
Time (Hr)

(**d**)

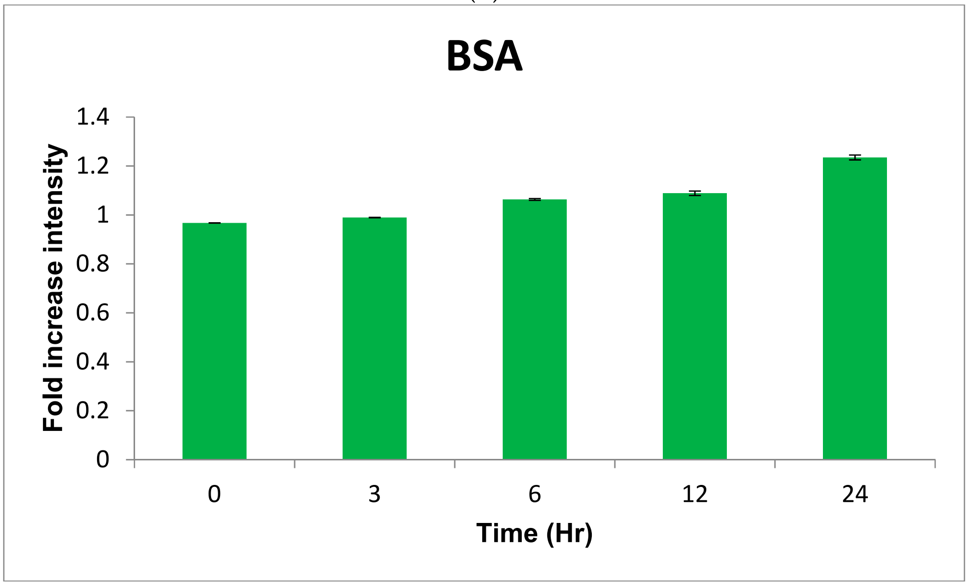
BSA
Fold increase intensity
1.4
1.2
1
0.8
0.6
0.4
0.2
0
0
3
6
12
24
Time (Hr)

(**e**)

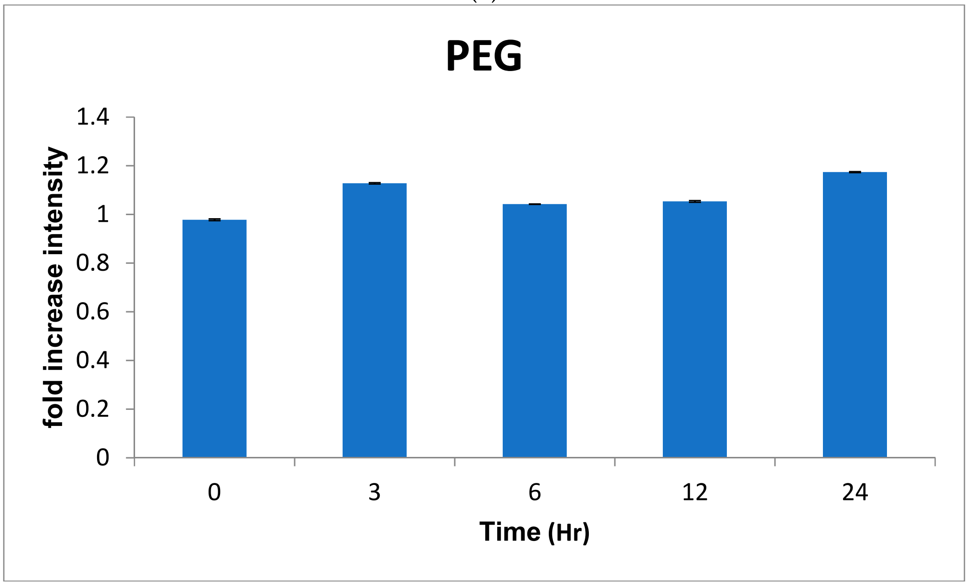
PEG
fold increase intensity
1.4
1.2
1
0.8
0.6
0.4
0.2
0
0
3
6
12
24
Time (Hr)

(**f**)

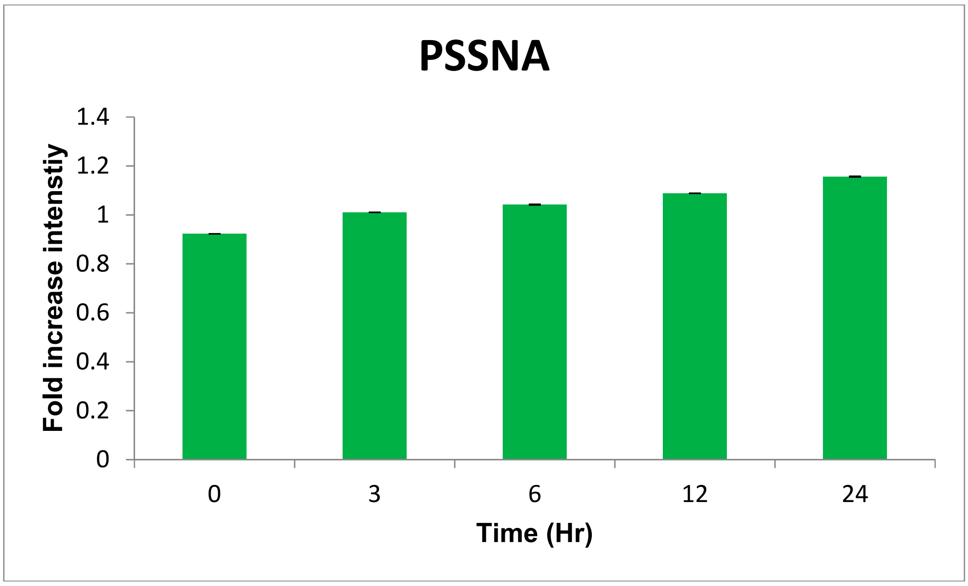

(**g**)

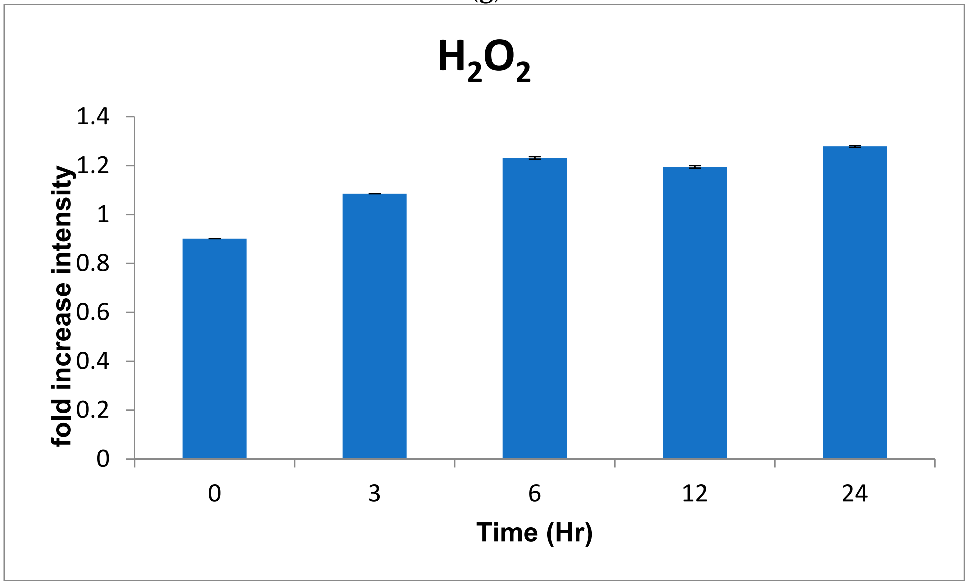

(**h**)

Figure S2. Graphs of the fold increase of apoptosis obtained from the ApoPercentage assay for the various GNP-ligands. (**a**) Fold increase of apoptosis for GNP-GSH. The apoptotic induction time-point for GNP-GSH was 3 hrs after which the cells recovered before apoptosis increased again due to depleted cellular nutrients. (**b**) Fold increase of apoptosis for GNP-PVP, (**c**) Fold increase of apoptosis for GNP-MUA, (**d**) Fold increase of apoptosis for GNP-Citrate, (**e**) Fold increase of apoptosis for GNP-BSA, (**f**) Fold increase of apoptosis for GNP-PEG, (**g**) Fold increase of apoptosis for GNP-PSSNA, (**h**) Fold increase of apoptosis for hydrogen peroxide (H_2O_2).

References

- ABCAM. 2021. How to convert between different concentrations [Online]. Available: https://www.abcam.com/help/how-to-convert-between-different-concentrations [Accessed date: 17.01.2022].
- Bueno, V. B. & Petri, D. F. S. Xanthan hydrogel films: Molecular conformation, charge density and protein carriers. *Carbohydr. Polym*. **2014**, *101*, 897–904.
- Gans, E. H., Chavkin, L. The use of polyethylene glycol in tablet coating. *J. Am. Pharm. Assoc.* **1954**, 43, 483–485.
- Nadi, M. M., Ashrafi Kooshk, M. R., Mansouri, K., Ghadami, S. A., Amani, M., Ghobadi, S., Khodarahmi, R. Comparative Spectroscopic Studies on Curcumin Stabilization by Association to Bovine Serum Albumin and Casein: A Perspective on Drug-Delivery Application. *Int. J. Food Prop*. **2014**, *18*, 638–659.
- Nair, B. Final Report On the Safety Assessment of Polyvinylpyrrolidone (PVP). *Int. J. Toxicol.* **1998**, *17*, 95–130.

- National Center For Biotechnology & Information. 2015. 11-Mercaptoundecanoic acid [Online]. Available: https://pubchem.ncbi.nlm.nih.gov/compound/543502 [Access Date: 27.03.2005].
- Okumura, M., Shimamoto, S., Nakanishi, T., Yoshida, Y.-I., Konogami, T., Maeda, S., Hidaka, Y. Effects of positively charged redox molecules on disulfide-coupled protein folding. *FEBS Lett.* **2012**, *586*, 3926–3930.
- Shovsky, A., Varga, I., Makuska, R., Claesson, P. M. Adsorption and solution properties of bottle-brush polyelectrolyte complexes: effect of molecular weight and stoichiometry. *Langmuir* **2012**, *28*, 6618–6631.
- Simonian, A. L., Revzin, A., Wild, J. R., Elkind, J., Pishko, M. V. Characterization of oxidoreductase–redox polymer electrostatic film assembly on gold by surface plasmon resonance spectroscopy and Fourier transform infrared–external reflection spectroscopy. *Anal. Chim. Acta* **2002**, *466*, 201–212.
- Tolwani, A. J., Campbell, R. C., Schenk, M. B., Allon, M., Warnock, D. G. Simplified citrate anticoagulation for continuous renal replacement therapy. Kidney Int **2001**, *60*, 370–374.
- Townsend, D. M., Tew, K. D.,Tapiero, H. The importance of glutathione in human disease. *Biomed. Pharmacother*. **2003**, *57*, 145–155.
- Venkatesan, R., Pichaimani, A., Hari, K., Balasubramanian, P. K., Kulandaivel, J., Premkumar, K. Doxorubicin conjugated gold nanorods: a sustained drug delivery carrier for improved anticancer therapy. J. Mater. Chem. B **2013**, *1*, 1010–1018.
